# Community health workers programme in Luanda, Angola: an evaluation of the implementation process

**DOI:** 10.1186/1478-4491-12-68

**Published:** 2014-12-09

**Authors:** Camila Giugliani, Bruce Bartholow Duncan, Erno Harzheim, Antônio Carlile Holanda Lavor, Míria Campos Lavor, Márcia Maria Tavares Machado, Maria Idalice Barbosa, Vera Joana Bornstein, Ana Lúcia Pontes, Daniela Riva Knauth

**Affiliations:** Social Medicine Department and Post-Graduate Programme on Epidemiology, Federal University of Rio Grande do Sul, Rua Ramiro Barcellos, 2600/419, Porto Alegre, RS Brazil; Oswaldo Cruz Foundation (FIOCRUZ), Fortaleza, Ceará Brazil; UNICEF and the Ministry of Health in Angola, Luanda, Angola; Community Health Department, Federal University of Ceará, Fortaleza, Ceará Brazil; Joaquim Venancio Politechnical Health School, Oswaldo Cruz Foundation (FIOCRUZ), Rio de Janeiro, Brazil

**Keywords:** Community health workers, Primary health care, International cooperation, Angola, Brazil, Agentes Comunitários de Saúde, Atenção Primária à Saúde, Cooperação Internacional, Brasil, Angola

## Abstract

**Background:**

The Community Health Workers (CHWs) Programme was launched in Luanda, Angola, in 2007 as an initiative of the provincial government. The aim of this study was to assess its implementation process.

**Methods:**

This is a case study with documental analysis, CHWs reports data, individual interviews and focus groups.

**Results:**

Until June 2009, the programme had placed in the community 2,548 trained CHWs, providing potential coverage for 261,357 families. Analysis of qualitative data suggested an association of CHWs with improvements in maternal and child access to health care, as well as an increase in the demand for health services, generating further need to improve service capacity. Nevertheless, critical points for programme sustainability were identified.

**Conclusions:**

For continuity and scaling up, the programme needs medium- and long-term technical, political and financial support. The results of this study may be useful in strengthening and reformulating the planning of the CHWs programme in Luanda and in Angola. Moreover, the lessons learned with this experience can also provide insight for the development of CHWs programmes in other parts of the world. By means of cooperation, Brazil has supported the implementation of this CHWs programme and can potentially contribute to its improvement.

**Electronic supplementary material:**

The online version of this article (doi:10.1186/1478-4491-12-68) contains supplementary material, which is available to authorized users.

## Resumo

**Introdução:**

O Programa de Agentes Comunitários de Saúde (PACS) de Luanda iniciou em Luanda, Angola, em 2007, como iniciativa do governo provincial. O objetivo deste estudo foi avaliar o seu processo de implantação.

**Métodos:**

Realizamos um estudo de caso com análise documental, registros das fichas de avaliação dos ACS, entrevistas individuais e grupos focais.

**Resultados:**

Até junho de 2009, o PACS de Luanda chegou a 2.548 ACS treinados, proporcionando cobertura potencial para 261.357 famílias. Por meio dos dados qualitativos, observou-se associação do ACS com ampliação do acesso aos cuidados de saúde, principalmente de mães e crianças, e aumento da demanda nos serviços de saúde, gerando necessidade de maior capacidade de resposta. No entanto, pontos críticos em relação à sustentabilidade do programa foram identificados.

**Conclusões:**

Para sua continuidade e ampliação, o Programa precisa de suporte técnico e apoio político e financeiro a médio e longo prazo. Os resultados deste estudo podem ser direcionados para reforçar e reformular o planejamento das ações do PACS de Luanda e de Angola. Além disso, os aprendizados que partem desta experiência podem servir de subsídio para o desenvolvimento de programas de ACS em outras partes do mundo. Por meio da cooperação, o Brasil apoiou a implantação do PACS de Luanda e tem potencial para contribuir na sua evolução.

## Background

Angola is undergoing a period of reconstruction, after experiencing a protracted civil war that lasted until 2002. This historical context of violent conflicts has left a legacy of low rates of production and capital accumulation, of severe debt, and extreme dependency on foreign countries, all of which markedly influence the health situation of the Angolan people. Based on an economy that depends largely on a single product (oil), since the end of the war, Angola has been gradually rebuilding its society, including the health system [1].

Data from the World Health Organization (WHO) have suggested improvements in the health status of Angolans in recent years [2], following the gradual increase of investments made in the social sectors, including health and education. Despite this progress, these indicators reflect a situation of extreme vulnerability, with some of the most alarming figures in the world: life expectancy at birth of 51 years, maternal mortality rate of 450/100,000 and a child mortality rate (under 5 years) of 158/1,000 [2]. Even today, almost 50% of child deaths are caused by diarrhoea, pneumonia, or malaria [2]. The capital, Luanda, divided into seven districts, accounts for almost one third of the population of Angola. This agglomeration, caused in large part by the war, further exacerbates the health scenario, favouring the persistence of communicable diseases.

Angola has a National Health System which is meant to provide universal access to health care services for all the country’s population [3, 4]. However, the fragile structure of the system, which is facing reorganization, still dealing with the ravages of war, imposes significant barriers to its proper functioning. The shortage and poor distribution of human resources, especially in rural and suburban locations, further aggravates this situation. In this context, since 2006, the Ministry of Health has been engaged in a process of revitalization of Municipal Health Systems, in order to accelerate the reduction of maternal and child mortality through decentralized and accessible interventions [5]. The proposal reinforces the expansion and upgrade of primary health care (PHC) as a fundamental feature of the development of the health system, following the international strategic guidelines for the African continent [6, 7].

With the revitalization process, in 2007 the Community Health Workers Programme (referred to as PACS, acronym from the Portuguese) was launched in Luanda, as an initiative of the provincial government. For its initial implementation in six districts, the Provincial Department of Health (PDH) called for the support of Brazilian professionals, who worked as consultants through the United Nations Children’s Fund (UNICEF). Allied to these, other Brazilian institutions, such as the Federal University of Rio Grande do Sul (UFRGS), the Federal University of Ceará (UFC) and the Oswaldo Cruz Foundation (FIOCRUZ), supported the initiative through technical cooperation, developing research activities, and exchange experiences.

We believe that the cooperation between Brazil and Angola, in addition to keeping with current trends in South-South partnerships, was motivated by an appreciation of the Brazilian experience with community health workers (CHWs), acknowledged worldwide through studies that have revealed the contribution of these workers, along with family health teams, in reducing child mortality [8–11].

Recent studies have demonstrated the effectiveness of CHWs in improving maternal and child health internationally [12, 13], but the way programmes are implemented depends on the context of each country, including, besides the health situation of the population, complex factors, such as political, social, economic and cultural. Therefore, because of its complexity, the CHWs programmes’ implementation process deserves special consideration in the research scenario.

Thus, motivated by the launching of the programme and by the cooperation with Brazil, the present study aimed to evaluate the implementation process of the PACS-Luanda, in order to draw lessons learned and recommendations for the future.

## Methods

The present study is part of a broader cooperation project between Brazil and Angola with the objective of supporting the development of PHC in Angola. The fieldwork took place in Luanda over 2 missions, lasting 2 to 3 weeks each, in the period between May 2008 and June 2009.

We used the case study method, with the following as sources of evidence: legal and technical documents, records of evaluation sheets from CHWs, semi-structured interviews, and focus groups. The study of these sources made it possible to analyze the implementation of PACS-Luanda, evaluating provision indicators (human resources, structural and organizational aspects, work materials) in relation to their adequacy (the extent to which the programme objectives were met) [14, 15]. The different types of respondents in the individual interviews and focus groups (provincial managers, district coordinators for the CHWs, and CHWs), allowed us to investigate and understand different perspectives of the phenomenon being studied.

For documental analysis, we used documents on health policies in Angola and Luanda, especially those related to PHC, in addition to the legal documents. An additional file lists all the documents examined in this analysis (see Additional file 1).

For individual interviews and focus groups, we developed a script for each type of respondent. The focus group technique was applied to the CHWs, for the possibility of obtaining their testimony given the social interaction among participants [16]. Participants were selected by convenience, based on a call from municipal coordinators. Individual interviews were conducted with two provincial level managers, the UNICEF representative responsible for supporting the PACS-Luanda (considered as a provincial manager) and six district coordinators (one from each district). The six focus groups (one per district) gathered forty-eight CHWs in total (average of eight per group). The interviews and focus groups were all conducted in Portuguese by the main researcher (CG), who is Brazilian and a native Portuguese speaker. All focus groups were co-facilitated by a second researcher, who was in charge of recording and taking notes. The interviews lasted on average 40 minutes, while the focus groups lasted approximately 1 hour and 20 minutes each. All interviews and focus groups were recorded and later transcribed.

Interviews and focus groups broadly covered the following areas: objectives of the CHWs programme, view of the programme’s progress in the reality of the districts where it was implemented, positive aspects, critical points and difficulties of the programme (or of the CHWs' work), and perspectives about the continuity or suggestions to improve the programme. Additional aspects covered were: assessment of the programme’s progress as a health policy in Luanda (in interviews to managers); description of the coordinator’s work and feelings about it, view of the CHWs' work, and description of data collection procedures by CHWs (in interviews to coordinators); and description of the CHWs' daily work, pointing out positive aspects and limitations (focus groups with CHWs).

All records made by CHWs, available from December 2007 to December 2009, with data on the number of trained CHWs and families they followed up during the period, were entered.

### Analysis

Documental analysis was used to describe the implementation of the programme and to make comparisons between observed events and what was originally planned. Records from CHWs were entered in an Excel spreadsheet (Microsoft, Redmond, WA, USA) for descriptive analysis over time through frequencies and proportions. For interviews and focus groups, content analysis was performed [17, 18]. Building on the analysis axes, previously assembled for field research (such as the objectives of the PACS, positive results and difficulties), the collected material enabled the creation of categories and subcategories. This was followed by a review of discursive passages allocated in each category in order to correct inconsistencies [18]. Patterns of convergence and divergence were identified among different types of respondents, seeking to characterize their specificities [17, 18]. To assist in the organization of data, we used a software for qualitative data analysis (MAXqDA) [19].

### Ethical considerations

This study was approved by the Ethics Committee of the Federal University of Rio Grande do Sul (UFRGS) and authorized by the PDH in Luanda. All participants read and signed an informed consent form.

## Results

### PACS-Luanda implementation process, according to documental analysis

The PACS-Luanda was an initiative of the provincial government, which provided funding for an initial period of two years of a pilot project. The PDH-Luanda assumed responsibility for its implementation. The proposal was presented as the 'flagship' for the revitalization process in the province, with the primary objective of reducing maternal and child mortality.

One of the central elements of the deployment of the PACS-Luanda was the definition of clear objectives to guide the work of the CHWs according to the context. Thus, the work of the CHWs began focusing on pregnant women and children under five, with the plan to expand to other population groups, with new objectives as early goals were met. Another important point for the programme’s implementation was the CHW profile. As prerequisites, the CHWs had to be a resident of the community where they would work, be older than 18, have at least 8 years of schooling, and have a good relationship with the community. According to all sources studied, the expectation was that the CHWs would act as a 'connecting point' between the community and the health service. To start the programme, the agreement was that each CHW would follow up, on average, 100 families while receiving an incentive of 4,000 kwanza - the equivalent of 50 USD - per month to work 8 hours a week with no formal employment relationship.

Six districts, covering both urban and rural areas in the peripheral area of Luanda province, were chosen for the implementation of the PACS: Cacuaco, Cazenga, Kilamba Kiaxi, Samba, Sambizanga, and Viana. These districts were prioritized by the PDH because of their vulnerability. Programme implementation occurred gradually, not simultaneously in all districts. In organizational terms, the programme, while coordinated by the PDH, was bound to the local administrative structures of the district and neighbourhood.

The implementation process of the PACS began in March 2007, with the preparation of the coordinators, followed by the training of the first CHWs in Cacuaco district. An important feature of this process is that the implementation occurred concomitantly to the training of the CHWs. Such strategy facilitated two important aspects: the on-the-job training based on practical tasks, and the process evaluation focused on results according to the objectives previously outlined. The initial training, lasting 42 days, consisted of both theoretical and practical activities (10 and 32 days, respectively). The CHWs were selected from nominations made by the District Administration, along with the Communal Administration and the Neighbourhood Residents Committee, considering the requirements mentioned above.

The initial stages of deployment are given as follows: mapping and registration of families (first stage), identification and follow- up of pregnant women (second stage), and identification and follow-up of children under five years of age (third stage). In the second stage, the CHWs also began working with the promotion of water treatment with sodium hypochlorite and the use of mosquito nets for malaria prevention.

**Figure 1:**
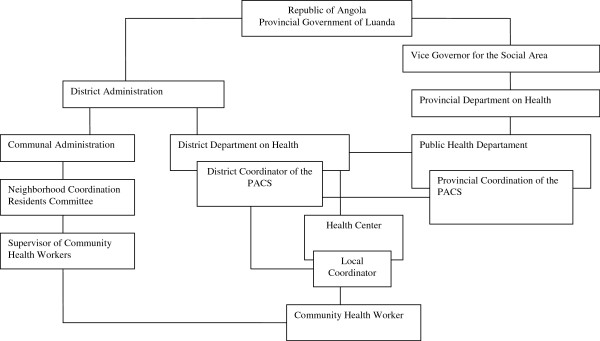
**Coordination structure of the Community Health Workers Programme (PACS) in Luanda.** Source: UNICEF Consultant’s Report, December 2007.

The coordination structure of the programme is presented in Figure 1. Each local coordinator would have at most 50 CHWs under their responsibility. During the implementation process, a new figure emerged, that of the supervisor, attached to the residents’ committee, to promote closer contact with the CHW.

For monitoring of activities, CHWs were instructed to fill in a record sheet that contained the following information: number of families/people being followed, number of children under five and of pregnant women being followed, number of pregnant women under adequate antenatal care, number of institutional deliveries, and information about: children’s vaccination status, breastfeeding, malnutrition, water treatment, and use of mosquito nets. Based on these records, regular meetings for evaluation were planned between the CHWs, supervisors, and coordinators (on a weekly basis in the first year of implementation, then in decreasing frequency), which were also planned as opportunities for ongoing training of the CHWs. The monitoring and evaluation data was then compiled at the district level and passed on to the provincial level.

The health system in Luanda functions through the coexistence of public and private services, with weak regulation and integration. Health posts and centres, in addition to municipal hospitals, the primary care points for the system, often suffer from poor communication with secondary and tertiary levels of care and are often still quite centralized at the provincial level in terms of management. Access to PHC services is often hindered by distance, poor responsiveness (lack of qualified personnel, tests, and drugs, for example) and the unauthorized charges for medical visits, which have become common within public services.

Regarding the integration of CHWs in this system, it was expected that the programme would be structured from a network of health facilities with multidisciplinary teams. The implementation project also foresaw that a reorientation of the health care model in order to expand and enhance access and resoluteness of PHC services would be fundamental. Thus, according to the documents searched, the PACS should have been placed in a wider context of political and institutional decisions.

The Brazilian consultants were directly involved in supporting the implementation process for twelve months, from May 2007, having been actively involved in conducting the training and organization of the structure of the PACS, and accompanying and supporting the managers. After this first year of work, they continued working through the broader cooperation project mentioned above, along with collaborators from other Brazilian institutions. The project established a direct partnership with the PDH in Luanda, the body responsible for coordinating the city’s PHC network, and with the local representation of UNICEF.

### Evolution of the coverage of the PACS

With the data available in the CHWs' assessment sheets, supplemented by other sources, we observed the evolution of the implementation of the programme in the 6 districts, reaching 2,548 trained CHWs and 261,357 families enrolled in June 2009 (Table 1). Data from CHWs who submitted their records were used as the basis for estimating the numbers stated for the total of trained CHWs. The exact number of active CHWs at that time could not be defined.

**Table 1:** **Community Health Workers Programme (PACS) numbers from December 2007 to December 2009, per district**

District (Commune and target population according to documents)	December	July	December	June	December
	2007 ^d^	2008	2008	2009	2009 ^d^
Cacuaco (Kikolo: 421,000 inhabitants)					
• CHW necessary to cover target population^b^	601	601	601	601	601
• Trained CHW	832	872	890	1094	1098
• Registered families	31,270	69,503	70,352	109,172	109,788
• Registered individuals	97,021	385,809	502,986	600,624	604,009
(% in relation to target population)	(23.0%)	(91.6%)	(119.5%)	(142.7%)	(143.5%)
Sambizanga (Ngola Kiluanji: 250,000 inhabitants)					
• CHW necessary to cover target population^b^	357	357	357	357	357
• Trained CHW	277	407	480	487	487
• Registered families	10,001	30,152	53,037	53,810	48,113
• Registered individuals	28,393	170,722	297,006	301,337	269,432
(% in relation to target population)	(11.4%)	(68.3%)	(118.8%)	(120.5%)	(107.8%)
Samba (Benfica: 108,850 inhabitants)					
• CHW necessary to cover target population^b^	155	155	155	155	155
• Trained CHW	11	59	100	150	131
• Registered families	1,261	6,913	11,717	17,575	13,223
• Registered individuals	4,380	24,003	40,683	61,025	45,914
(% in relation to target population)	(4.0%)	(22.1%)	(37.4%)	(56.1%)	(42.2%)
Kilamba Kiaxi (Golfe: 389,500 inhabitants)					
• CHW necessary to cover target population^b^	556	556	556	556	556
• Trained CHW	96	78	166	176	160
• Registered families	4,800	6,853	14,584	18,532	16,620
• Registered individuals	20,513	31,074	66,131	71,336	63,975
(% in relation to target population)	(5.3%)	(8.0%)	(17.0%)	(18.3%)	(16.4%)
Cazenga (Cazenga: 643,123 inhabitants)					
• CHW necessary to cover target population^b^	918	918	918	918	918
• Trained CHW	258	373	373	641	641
• Registered families	8,036	28,236	34,849	62,267	64,151
• Registered individuals	27,734	97,451	225,676	406,661	418,966
(% in relation to target population)	(4.3%)	(15.2%)	(35.1%)	(63.2%)	(65.1%)
Viana^a^ (Km 9 and Km 12: 600,000 inhabitants)					
• CHW necessary to cover target population^b^	857	857	857	857	857
• Trained CHW	120	-	339	360	131
• Registered families	8,037	-	33,900	36,000	32,311
• Registered individuals	40,186	-	169,500	180,000	161,555
(% in relation to target population)	(7.0%)		(28.2%)	(30.0%)	(26.9%)
Total of Luanda province (2,412,473 inhabitants^c^)					
• CHW necessary to cover target population^b^	3,444	3,444	3,444	3,444	3,444
• Trained CHW	1,594	1,789	2,348	2,548	2,517
• Registered families	63,406	141,658	238,699	261,357	251.895
• Registered individuals	218,227	709,060	1,301,982	1,440,983	1,402,295
(% in relation to target population)	(9.0%)	(29.4%)	(54.0%)	(59.7%)	(58.1%)

^a^The only available records for the district of Viana were extracted from the UNICEF consultant’s report of December 2007, presentations by the Provincial Department on Health in Luanda (December 2008 and December 2009), and from interview with district coordinator (June 2009), enabling a projection of the number of families and individuals covered by the programme.

^b^Estimated as follows: target population/700 (700 equals 100 (number of families for each CHW to follow-up) × 7 (projected mean number of individuals per family)).

^c^Province population equals the sum of all target populations for the six districts.

^d^In December 2007, the number of trained CHWs includes CHWs with unconcluded training, this explains the number of CHWs in the district of Kilamba Kiaxi being higher in December 2007 than in July 2008. The source of the data for December 2009 was a presentation of the Provincial Department on Health in Luanda (original CHW records were not available).

The figures reveal two major discrepancies, which are interrelated: between the number of CHWs planned and what was proving to be actually necessary to cover the population; and among the target population considered in planning the programme and the population found upon initiating the work of mapping and registration. For this reason, some districts had more than 100% coverage.

The potential coverage achieved for CHWs interventions, according to the number of trained CHWs, was 60%. It can be observed that, from June to December 2009, there was a reduction in the number of CHWs, which may be due to difficulties faced, generating dropouts, or to inconsistencies between sources of records.

The low level of education of CHWs, the flaws in supervision of CHWs after the initial training, the workload and accumulation of different functions are all factors contributing to generate large discrepancies in the records made by CHWs. Therefore, health indicators generated by CHWs, such as vaccination rates and attendance of prenatal care among pregnant women, could not be used because they were filled out irregularly and showed inconsistencies. So the progress of such indicators could not be measured.

### Profile of CHWs and coordinators

The majority of CHWs were studying or had other occupations. Some were unemployed nursing technicians, many with the plan of becoming nurses or doctors. Several were already involved with the neighbourhood residents committee or were working as activists in non-governmental organizations (NGOs). There was a slight predominance of men (56%).

Almost all municipal coordinators were nursing technicians working in the District Department of Health, taking on additional roles in the local health facility or engaged in university studies. Managers at the provincial level (two doctors and a nurse) also took on multiple roles. In only one district, the first to have the PACS implemented, the district coordinator had exclusive dedication to the PACS, explaining, in part, why this district had the largest number of trained CHWs and a relatively less irregular process of monitoring and evaluation.

### Positive aspects of the work of CHWs

The increased demand for health facilities was perceived as positive, despite having also been mentioned as a difficulty, because of the low capacity of health services. In the documents examined, it was mentioned that one of the main reasons for the increase in demand was the reduction in unauthorized charges for visits, and the importance of CHWs in sharing this kind of information with the coordination was clearly stated:

*‘Over the last year, the health centre did not have as much influx as it does today. With the activity of the community health worker in encouraging, breaking some taboos of the community (such as the allegation that there is charging and maltreatment at health centres), they were then able to educate the community with regards to seeking the nearest health facility.’* (District coordinator)

In all districts, both CHWs and coordinators reported increased demand for maternal and child health care:

*‘Pregnant women didn’t attend prenatal care, but now, with the community health worker, they know they have to go to the centre to monitor the health of the baby and mother, and also, before, the children were not getting vaccinated, but now they do.’* (CHW)

The importance of the records made by the CHWs was also highlighted, whether in the notification of diseases, or simply counting the people living in each micro-area, leading to improved access of the government to local data.

The CHWs working on the districts most affected by a major cholera outbreak in 2006 demonstrated the importance of their work in the prevention of new cases, especially through water treatment. Also, from the perspective of managers, CHWs were important in terms of preventing a new epidemic:

*‘From the moment that the community health workers began to work, cases decreased dramatically. I believe they had a very big role in the distribution of hypochlorite house-to-house and in the support to families with regard to hygiene, sanitation, guidance even for simple hand washing, in detecting cases quickly in order to get to health facilities as early as possible, rehydration of patients at the local level, at the level of people’s homes. I believe that this contributed greatly, so as to decrease the number of cholera cases and of home deaths.’* (Provincial coordinator)

Concerning the increased usage of health facilities, units were often not prepared to meet this increased demand, and for many of them it was not possible to hire more staff due to administrative barriers. In this regard, the provincial administration is planning to increase the capacity of health facilities, which would have more space, more professionals, and longer hours of operation. Some progress in the structure of primary care services, even if limited for the moment, was noted:

*‘In transforming the posts into health centres, we can then adjust the staff both in terms of numbers and quality. For example, in the district where the community health workers training was initiated, the health post was transformed into a health centre and this change has already enabled the hiring of two doctors, the deployment a delivery room: it is an upgrade.’* (Provincial coordinator)

In the discourse of managers, the importance and specificity of the work of CHWs appears in their proximity to the community, justifying the investment in the continuity of the PACS.

*‘The community health worker he's there, he will see, because the caregiver* (*at the clinic*) *does what? He gives orientation, but the mother goes home, and who will then continue to observe, monitor what is being done is the CHW. So it is a key point for us, he will give us what we cannot see when we are at the clinic and I think it's a pretty good bet if we continue to invest in the community health worker.’* (Provincial coordinator)

### Barriers identified

The most mentioned difficulties, which also appear in the conclusions and recommendations of PACS documents, were the delay of payment to the CHWs and the lack of material to fulfil the tasks of one's job.

The delay of the monthly incentive was mentioned in all districts, both by CHWs and coordinators, appearing as a greater problem than the monetary value itself. Late payments, as well as the lack of a formal contract, undermined the dedication of time to work and led to many withdrawals:

*‘The delay in paying our incentives is too much, it’s already not too much and it’s not worth it, and so with this delay, there are even people who are quitting. For people to enhance their desire and strength to work, the incentive needs to improve, and to be paid on time, even if the amount is little, if we receive it every month, we will work in our communities with good will.’* (CHW)

*‘Many end up quitting,* ( *…* ) *when they find a better job that will occupy the free time he had, for example, he does not think twice, because he will have a better job and will also have a greater pay.’* (Provincial coordinator)

The lack of materials for the job, such as mosquito nets, hypochlorite to treat water, or boots for walking in wet weather creates conflicts of trust between CHWs and households, who claim to have their needs unassisted:

*‘When you make promises and do not deliver on these promises, this begins to discredit our work. “Give me a mosquito net?” “No, next week we’ll bring” successively and we start to lose that confidence from our families.’* (CHW)

There were also clear feelings of demoralization among CHWs with regards to the difficulties encountered in health facilities, including charges:

*‘The consultation is free, so the* (*community health*) *workers go and explain to their families, but what happens is that when the* (*community health*) *worker turns his back on the families, they are being charged, so there is a discrepancy.’* (District coordinator)

There was some ambiguity regarding the relationship between CHWs and other health workers in the health facilities. Often these relationships were marked by fragile bonds between the two, which, when not due to an ignorance of the responsibilities of the CHWs on the part of the health workers, could be the result of an unwillingness to accept CHWs and their roles in the health units. In this sense, CHWs were seen as individuals who would 'disturb the environment' by creating 'more work', witnessing possible incidents of unauthorized consultation charges, and generally acting as a 'stone in one’s shoe'.

Another difficulty encountered in the daily work of the CHWs was that they often referred individuals to health units who may not have had the money for transportation to get there. Thus, the CHWs, despite having performed his or her job, would sit helplessly by the ineffectiveness of his or her own orientations. The CHWs also expressed that their work was isolated from other public services such as garbage collection, for example:

*‘People complain to us “ah, you came, talked about the garbage, we have to do this, but here on our street there is no container for collection, we do not have electricity nor running water, the water doesn’t reach here”.’* (CHW)

Finally, the overload of coordinators, both at the provincial and district levels, was identified as a difficulty, placing the importance of having human resources with more time availability to dedicate to the programme.

### Challenges and perspectives

The sustainability of the programme, especially in financial terms, was raised by the managers as the biggest challenge, along with the need to create an institutional link for the CHWs:

*‘Another problem that is more serious is the affiliation of the* (*community health*) *worker. So far we do not know, it is a project, the project usually has a beginning and has an end, but we see that this programme is not supposed to end. Hence the need for a sustainable programme and this is a critical situation yet, so far this is a project of the provincial government, it is the government that is funding the costs of training and incentives, and we do not yet have our own mechanisms for expediting these funds.’* (Provincial coordinator)

Clearly, this stands as critical to the continuity of the programme because, in practice, there is a financial gap between the pilot programme and its expansion and consolidation. As a possible alternative, there are prospects of framing the PACS within the administrations at the district level, facilitating accountability for maintaining the programme at the local level. Decentralization of health management appears as a fundamental discussion in terms of health planning across all documents analyzed, being suggested as a way for improving dynamism and quality of local actions.

Regarding evaluation of the programme, its importance was evident in the case of Luanda. Provincial managers were concerned about the demonstration of results, so that the programme could attract political support and investment. The province of Luanda is being requested to support the implementation of the PACS in other provinces; however, there remains the need to first affirm the programme where it started.

The initial project included the incorporation of tools for monitoring and evaluation on a permanent basis. These tools were introduced, but their operation had several flaws, such as inappropriate completion of records, lack of systematization and computerization, and communication difficulties within coordination structure. Nevertheless, health officials recognized the potential of CHWs in collecting reliable data, due to their close follow-up to families.

Regarding training, CHWs indicated that the initial training was adequate in terms of content and format, but that continuous updates, such as regular refresher courses, were necessary. This need was also expressed by the managers interviewed. Finally, we identified the need to strengthen the integration of CHWs within health units, which had been a goal of the programme from the beginning according to its project.

## Discussion

The evaluation of the implementation process of the PACS-Luanda allowed for the linking of some contextual characteristics to the described results. Despite the irregular implementation process, the programme has provided initial training for a large number of CHWs, which, according to qualitative analysis, was associated with increased demand for health services and increased access to health care, especially among mothers and children. However, several difficulties, such as unavailability of resources and lack of institutional affiliation of the CHWs, appeared as limiting factors. In fact, the implementation of such a complex intervention, a CHWs programme, is influenced by several contextual factors [20]. In the case of Luanda, we highlight some of them: fragmentation of the health system, the fragility of social policies in general, centralization of management, shortage of skilled health workers, and urban agglomeration.

Some aspects of the PACS-Luanda can be compared with CHWs programmes in other parts of the world. The predominance of men may be related, as well as to cultural issues, to high unemployment and limited availability of public sector jobs, a situation that has been reported in other countries (India and Zambia, for example) [21]. In some African countries, in India and in Brazil, unemployment and job search has been described as motivation to work as a CHW [21–23].

When making comparisons, it is important to highlight that in Brazil the PACS was developed in the broader context of health reform that gave rise to the Unified Health System (SUS - the Brazilian acronym for *Sistema Único de Saúde*), which brought about great improvements in terms of access to health care [24, 25]. This framework within the wider health system was crucial to the success of the PACS in Brazil. Through the results achieved in the field of maternal and child health, the programme quickly gained support from the government, which determined its expansion to the whole country and the creation of the Family Health Programme [26]. This seems to be one of the major differences from the PACS-Luanda, which arises in a context where the health system is still characterized by fragmentation and deterioration resulting from war, where the resources of the state are mixed with those of international agencies, and still largely allocated to vertical programmes. Considering these contextual peculiarities, the building of SUS in Brazil emerges as a great heritage to be shared in an international cooperation initiative.

A recent international review has shown that large-scale public sector CHWs programmes are complex entities that require adapting a systems perspective to the national and local contexts and that proper costing of such programmes and assurance that those costs can be paid for on a sustainable basis are essential for its effectiveness [27]. Thus, considering the complex chain of factors involved in the success of CHWs programmes, it is important to outline that the outcome of this paper - an analysis of the implementation process in Luanda - may be useful to other countries, especially those that have historical and political similarities with Angola. One example is Rwanda, where a genocide in 1994 has decimated the country’s fragile economic base, destroyed a large share of its human capital, and eroded the health infrastructure. Years later, Rwanda has gone through sharp economic growth, and has made remarkable progress in primary health care, implementing CHWs, amongst other measures. Besides having donor assistance contributing to a large share of the national health budget, one important difference in Rwanda was that the Ministry of Health has managed to direct donors to align their contributions with national policies [28].

The fragile bond of the CHWs with the health unit found in Luanda has also been described elsewhere: in Brazil, in the beginning, the incorporation of the CHWs in the health team posed a threat to nursing assistants, who were unaware of their role. Then, with the recognition of CHWs, the responsibilities attributed to them became somewhat exaggerated [29]. Concerning this same issue, an international review stated that health professionals often appreciated the contribution of CHWs in reducing their workload, but some of them thought that CHWs actually added to their workload and feared a loss of authority [30]. Regarding the integration of CHWs with the facility-based health workers, international publications [31, 32] have identified a formal relationship with the health service as a key determinant of the effectiveness of CHWs programmes.

The lack of provision of adequate work materials was also a main impediment to the success of the PACS-Luanda. Indeed, other studies have shown that a lack of adequate supplies and frequent depletion of necessary stock are major barriers to effective implementation in primary care settings [33]. However, it is recognized that the provision and availability of human resources and commodities need to be coupled with sound governance, assurance of the supply chain, and attention to quality-of-care issues [33].

In Luanda, CHWs considered their initial training adequate, but insufficient. In Iran, provision of a comprehensive training programme, including both initial and continuing training, enabled the CHWs (called *behvarzes*) to provide comprehensive care with an emphasis on disease prevention and health promotion. The PHC system in Iran recognizes in-service training of a *behvarz* at regular intervals - workshops, monthly meetings, and refresher courses aim at integrating new policies and changes into the current work [34].

In Angola, we identified a clear trend towards decentralization of the management of PHC services, characterized in the literature as a need and a great opportunity for ongoing improvements in the health system in this country [35]. Moreover, the trend also appears in the guidelines of international agencies involved in supporting the development of the health system in Angola [7]. In the context of the PACS-Luanda, decentralization could present itself as a good opportunity to affiliate the CHW locally and to bring about a closer relationship between CHWs and facility-based health workers, thus enhancing the response to local needs. In Brazil, a study about the beginning of the PACS in Ceará showed that the autonomy of municipalities was an important feature in responding to local needs and gaining legitimacy [24]. However, while decentralization may facilitate management and adequacy of programmes, it can also generate inequities and disarticulation with the central level. In addition, it requires massive investment in human resources development at the district level.

This study highlights the need to incorporate effective strategies for evaluating the PACS-Luanda. In Brazil, the results of the evaluations that have been conducted since the beginning of the PACS generated huge political investment at the time, so they were crucial for the scaling-up of the programme, and remain a fundamental element for its ongoing improvement [8, 24, 26, 36]. In the international literature, evaluation serves as a key tool for demonstrating impact, predicting costs, and documenting the factors associated with the successes and failures of the programme [31]. A recent review showed that monitoring and evaluation, with adjustments to the programme based on these findings, is essential both for effective scale-up and long-term programme effectiveness at scale [27]. In the case of Luanda, where low level of schooling of CHWs was identified as a barrier to obtain data and maintain reliable records, we raise the point that years of schooling (as a prerequisite for CHW selection) might not be sufficient to determine a level of educational achievement necessary to fulfil the duties of satisfactory data collection. In the studied context (Angola), the fact of having eight years of schooling does not necessarily mean a good educational level. This is largely a consequence of deterioration of the quality of education during the years of war, which can still be perceived. Thus, in Angola and in similar contexts, additional information about abilities of CHWs in terms of reading and obtaining data, and in filling out forms, for example, could be helpful in the process of their selection and training.

From these comparisons, particularly with Brazil, and under the initiative of cooperation for the development of PACS-Luanda, it is essential to reflect on the processes of 'transfer' and 'translation' of experiences to different contexts, assessing how contributions can be adapted and made more effective. Previous international studies have shown that the work of CHWs should reflect the circumstances of their context, in terms of needs and resources, for example, requiring flexibility throughout the implementation process [21]. We agree and, furthermore, argue that it is also necessary to find more effective ways to turn research into policy and actions [37], enabling managers and others responsible for developing policies to make use of research findings.

In general, the international literature, that has assembled lessons learned from experiences with CHWs since the 1960s, aligns with the critical points made in the case of Luanda [21, 23, 27, 31, 32]: training only is insufficient, supervision and continuous support are required; CHWs' tasks must be clearly specified; a sound remuneration system must be in place; the programme should be framed within a wider context of political support which guarantees sustainability; and it is necessary to evaluate its effectiveness. In the same vein, a comprehensive review has offered some conclusions about CHWs programmes: they can improve access to essential health care, especially for children; CHWs must be properly selected and trained and receive ongoing support and supervision to achieve good results; many CHWs programmes failed by not properly considering in the planning stage the efforts and costs required for the proper functioning of the programme; the communities must have ownership of the programme and be able to support the CHW in their actions; and there is strong evidence to suggest that the CHW should be a remunerated worker [38]. Thus, well-designed, functional support and interaction between CHWs and health systems are essential for effective community health services [27]. Various elements of the case of Luanda are in agreement with these conclusions. Additionally, a review of qualitative evidence to assess the factors affecting the implementation of CHWs programmes for maternal and child health showed that important facilitators include regular and visible support from the health system and the community, and appropriate training, supervision and incentives [30]. In terms of measures to be implemented globally, the international community agrees that the development of the health workforce at the primary health care level, according to national circumstances and needs, while acknowledging the importance of technical cooperation and capacity-building, should be prioritized to enhance equity in access [39].

It is important to consider some limitations of the present study. CHWs who participated in the focus groups are those who were contacted by their supervisors for being more participatory. Thus, it is possible that the difficulties faced were greater and even different from those reported in this study. The lack of reliable statistics, a problem for Angola as a whole, prevented the analysis of the evolution of health indicators throughout the implementation of the programme. Although it is not possible to generalize our findings, given the nature of the study, we believe that the analysis of this particular situation can teach us about the phenomenon of the implementation of a CHWs programme in the context of an international cooperation initiative. Another limitation is that inconsistencies and incompleteness of CHWs' records prevented us from measuring some basic process indicators, such as the number of children under five and pregnant women being followed by CHWs.

It is worth mentioning that the participation of researchers in this study occurred in conjunction with their involvement in the implementation process itself, through cooperation, which began with the consultants from UNICEF. As the study aimed at supporting the development of PACS-Luanda, there was no intention of making an entirely external or independent evaluation. On the other hand, an advantage of this approach was the proximity to decision-makers, which can be considered strategic for the purposes of such an evaluation.

Further details are available in Additional file 2, a short movie about the implementation of the PACS-Luanda and the cooperation between Angola and Brazil in this context.

## Conclusions

As reflections and final considerations, we point out the following:

-  It is important, in the case of Luanda but also in general, to strengthen conditions related to the broader political context, which appear to be crucial to the success of the PACS: the programme’s integration in the health system and in the context of broader social policies in order to deal with problems such as poverty and sanitation. The progress in building a democratic state is also crucial for the development of public policies
-  The proper costing of the PACS, considering there is an intention to scale-up the programme, and assurance of funds on a sustainable basis are essential for an effective large-scale CHWs programme
-  The PACS-Luanda began with a priority focus on maternal and child health with the rationale that a reduction in mortality among this population group could aid in providing enhanced visibility to the initiative. This was the path taken in Brazil, and it may be a good opportunity for the beginning of a more comprehensive PHC strategy
-  The increased demand for health care services, which was felt as a difficulty by health facilities, can also be seen as an incentive to invest in the network of PHC services and increase its capacity to respond to people’s needs. The idea is that the services can be enhanced to provide ongoing support and continuous supervision to the CHW
-  The CHW should not be a volunteer worker in this context. Appropriate payment, consistent with full-time dedication, increase motivation and improve workers’ performance. Therefore, it is essential to seek ways to formally affiliate CHWs to the health system
-  In order for CHWs to be motivated and qualified for their positions, it is necessary to invest in their training and it is important that any competent institution bears this responsibility
-  The PACS can contribute to data collection for the evaluation and planning of health actions in Angola. CHWs' records may be an important source for vital statistics, as it was for the Brazilian northeast and north in the first decade of the implementation of the PACS there
-  The overburden of the programme coordinators, with numerous responsibilities and accumulation of different functions, is an obstacle to the smooth functioning of the programme. As there are still relatively few people with university level training in Angola, these eventually take management positions and typically are responsible for two or more areas. Even taking into account that this is not a reversible scenario in the short term, it is important to highlight the need for professionals who are able to devote more time to the programme
-  In the context of cooperation, we consider that the PACS-Luanda, having been an initiative of the Angolan government, has a greater probability to succeed, if compared with initiatives that come from international agencies or organizations, in terms of autonomy and sustainability

Finally, the introduction of the PACS-Luanda enabled the creation of a large number of trained CHWs and the coverage by the programme of a large number of households. However, the implementation process was irregular and presented many shortfalls, which appeared to threaten its sustainability. For its continuity and expansion, the programme requires technical support and political and financial backing in the medium and long term.

The Brazilian experience shows the contribution of the PACS to promote citizenship and social justice, based on the building of health care models that assure health as a right. Despite the differences between the Brazilian and African historical contexts, citizenship is a central element for democracy in any country. Building health systems that are oriented to primary health care and health promotion is related with the construction of citizenship in society as a whole. In Angola, citizenship can be considered and addressed as a strong tool for building peace beyond the absence of war. Therefore, the PACS can contribute greatly to building active citizenship, to the extent that it promotes within the population the notion of health as a right through its educational activities [40].

The findings presented here can be directed to strengthen and reform the planning of the PACS-Luanda, through rethinking or reformulating some contents of programme planning and design, utilized tools, CHW training, supervision, monitoring of activities, and evaluation. Moreover, they can eventually be considered in conceiving a national CHW policy in Angola. Finally, the lessons learned in this study can also be used to reflect upon the current challenges of the PACS in Brazil and the role of international cooperation in health.

## Authors’ information

CG has been involved with cooperation work in Angola since 2005, first in rural areas with the NGO Médecins du Monde, and later in Luanda, for the support of primary health care policies, especially those involving community health workers. ACHL and MCL were involved with the creation of the community health worker’s programme in Brazil, in the 1980s, and their advice and consultancy was requested in 2007 by the Government of Luanda to support the implementation of the community health worker’s programme there.

## Electronic supplementary material

Additional file 1: **Documents examined for the documental analysis.** Description of data: this file lists all the documents that were examined for documental analysis. (DOC 42 KB)

Additional file 2: **'Angola with us' short movie.** Description of data: this short movie (duration: 19 minutes) is about the implementation of the Community Health Workers Programme in Luanda and the cooperation between Angola and Brazil in this context. (DOCX 13 KB)

## Abbreviations

CHWs: community health workers
FIOCRUZ: Oswaldo Cruz Foundation
NGO: non-governmental organization
PACS: acronym from the Portuguese, referring to Community Health Workers Programme
PHC: primary health care
PHD: Provincial Department of Health
SUS: acronym from the Portuguese, referring to Unified Health System
UFC: Federal University of Ceará
UFRGS: Federal University of Rio Grande do Sul
UNICEF: United Nations Children’s Fund.
