# Supplementary material for: Community health workers programme in Luanda, Angola: an evaluation of the implementation process
Source: Hum Resour Health. 2014 Dec 9;12:68. doi: 10.1186/1478-4491-12-68 (PMC4292814; doi:10.1186/1478-4491-12-68)
Supplement: Supplementary file 1 — Additional file 1: Documents examined for the documental analysis. Description of data: this file lists all the documents that were examined for documental analysis. (DOC 42 KB) [file 12960_2014_467_MOESM1_ESM.doc]

Additional file 1: Documents examined for the documental analysis

| **Title of document**  **(Institution and date)** | **Description of document** |
| --- | --- |
| Constitution of the Republic of Angola  (National Assembly, January 2010) | The Constitution is the Supreme and Fundamental Law of the Republic of Angola. |
| Presentation of evaluation data of the PACS  (PDH Luanda, 2009) | Data presented by the provincial coordinator, during her visit to Brazil, with the results achieved by the PACS by December 2009. |
| National Health System on the District Level (4th draft)  (Angola Ministry of Health, October 2009) | Strategic and operational tool to guide and follow-up the district and provincial managers in the process of planning and managing the districts’ health systems, with the goal of improving the population’s health status, responding to its expectations, and seeking to create a financial sustainability. |
| National Health Policy (5th draft)  (Angola Ministry of Health, March 2009) | Defines the main guidelines that should be implemented through the National Plans of Sanitary Development and Operational Plans, establishing the strategic orientations for improving the population’s health status and quality of life. |
| Results of the evaluation meeting 2008  (PDH Luanda, December 2008) | Data presented by the provincial coordinator with the results achieved by the PACS by December 2008. |
| Revitalization of District Health Services  (Angola Ministry of Health, January 2008) | Presents strategies to strengthen the implementation of the Strategic Plan to Accelerate the Reduction of Maternal and Child Mortality (2004-2009) |
| Plan of Sanitary Development of Cacuaco District (2008-2011)  (UNICEF, June 2008) | Presents the action plan for the revitalization of health services in Kikolo commune, Cacuaco district (2008-2009) |
| Handbook for the training of community health workers  (UNICEF and PDH Luanda, 2008) | Describes in detail the contents, activities and pedagogical methods for the CHWs’ training course |
| Guide for the training of CHWs coordinators  (UNICEF and PDH Luanda, May 2008) | Describes the contents for the training of CHWs coordinators, as well as the main tasks of CHWs. |
| PACS Consultancy Report  (UNICEF, February 2008) | Report on the development of the program until the indicated date, and recommendations for continuity. |
| PACS Consultancy Report  (UNICEF, December 2007) | Report on the development of the program until the indicated date, and description of next steps. |
| PACS Consultancy Report  (UNICEF, August 2007) | Report on the development of the program until the indicated date, and description of next activities. |
| PACS Consultancy Report  (UNICEF, July 2007) | Report on the development of the program until the indicated date, and description of next activities. |
| PACS Consultancy Report  (UNICEF, June 2007) | Report on the development of the program until the indicated date, and description of next activities. |
| PACS Consultancy Report  (UNICEF, May 2007) | Report on the first activities of the consultancy and impressions. |
| Community Health Workers Program of Luanda “Vigilants of Health”  (PDH Luanda, January 2007) | Document containing the initial proposal for the deployment of the PACS. |
| Law of Bases of the National Health System  (Journal of the Republic, August 1992) | Defines the principles and general directions of health policies, in its specific articles |

Abbreviations: PACS: Community Health Workers Program, acronym from the Portuguese; PDH: Provincial Department of Health; UNICEF: United Nations Children’s Fund ; CHWs: community health workers.
