# Supplementary material for: Community health workers programme in Luanda, Angola: an evaluation of the implementation process
Source: Hum Resour Health. 2014 Dec 9;12:68. doi: 10.1186/1478-4491-12-68 (PMC4292814; doi:10.1186/1478-4491-12-68)
Supplement: Supplementary file 2 — Additional file 2: 'Angola with us' short movie. Description of data: this short movie (duration: 19 minutes) is about the implementation of the Community Health Workers Programme in Luanda and the cooperation between Angola and Brazil in this context. (DOCX 13 KB) [file 12960_2014_467_MOESM2_ESM.docx]

This is the link to access freely the short film “Angola with Us, Community Health Workers”.

The MPEG4 file could not be uploaded due to size limit.

http://vimeo.com/66918749
